# Editorial: Mechanisms of Cell Adhesion in Hematopoietic Stem Cells

**DOI:** 10.3389/fcell.2021.826554

**Published:** 2021-12-21

**Authors:** Shinobu Matsuura, Alessandra Balduini, Maurizio Onisto

**Affiliations:** ^1^ Whitaker Cardiovascular Institute, Cardiovascular Section, Department of Medicine, Boston University School of Medicine, Boston, MA, United States; ^2^ Department of Molecular Medicine, University of Pavia, Pavia, Italy; ^3^ Department of Biomedical Engineering, Tufts School of Engineering, Tufts University, Medford, MA, United States; ^4^ Department of Biomedical Sciences, School of Medicine and Surgery, University of Padua, Padua, Italy

**Keywords:** hematopoietic stem cell, stem cell niche, adhesion, extracellular matrix, laminin, acute myeloid leukemia, leukemia stem cell, tetraspanin

Four decades after Lord et al. (1975) and Schofield (1978) proposed the concept of a specialized microenvironment in bone marrow in support of hematopoietic stem cell function (Scadden, 2014), the field is more active and vibrant than ever. Seminal works contributed to define the elements that constitute the hematopoietic stem cell niche, or, perhaps, niches: accumulated evidence suggests that the location of the bone marrow hematopoietic niche is perivascular, with a possible contribution of the bone endosteal space. Supporting structures in those environments include cells (endothelial cells, perivascular mesenchymal stromal cells, megakaryocytes, sympathetic neurons and Schwann cells, etc.), bone and bone lining cells (osteoblast, osteoclast) and extracellular matrix (fibronectin, collagen, osteopontin, hyaluronan, tenascin, etc.). As the physical components of the niche are defined, new questions come to the fore: what the molecular mechanisms involved in HSC adhesion are; whether, and by what mechanisms, adhesion may control HSC stemness, proliferation and differentiation; how adhesion mechanisms in HSC differ from those in more mature progenitors or terminally differentiated cells; what disturbances in adhesion are observed in disease states; and whether we can manipulate stem cell function through manipulation of adhesion. In this topic, we present a lineup of exciting and impactful publications responding to these questions. Approaching the questions from different angles and perspectives, the original publications and reviews presented here provide novel data and insightful information that will inspire the reader and will lead the way to future discoveries in the field.

Laminin is an extracellular matrix adhesion-protein present in the basement membrane of most vascular structures in bone marrow, including sinusoids and arteries (Coutu et al., 2017). Godavarty et al., using human CD34^+^ cells, examine the role of two novel laminin receptors, α7β1 and basal cell adhesion molecule/Lutheran (BCAM/Lu), in hematopoietic stem cell biology. BCAM/Lu, which interacts exclusively with laminin has so far only been detected in the erythroid lineage (De Grandis et al., 2013), but not on early hematopoietic stem and progenitor cells (HSPC).

Acute myeloid leukemia (AML) is a heterogeneous, complex, and deadly disease. Therapy is still highly reliant on intensive chemotherapy, which is efficient in reducing the bulk of leukemic cells, but is not effective at eliminating the leukemia stem cells (LSC). The presence of residual LSC is believed to be responsible for relapse and chemotherapy resistance; hence, the urgent need for therapies that can specifically target the LSC. Mirroring their healthy counterpart, LSC are nested in specialized niches which support their stemness, competition advantage, and acquisition of resistance to therapy. Villatoro et al. provide a comprehensive review of the most recent advances in the understanding of LSC niches, and provide an overview of clinical trials aiming at targeting these processes for AML treatment. Erbani et al. report on the important novel discovery of the role of CD162, receptor of E-selectin, as a key AML cell surface receptor involved in AML progression, bone marrow retention and chemo-resistance. Their findings highlight specific blockade of AML cell surface CD162 as a potential novel niche-based strategy to improve the efficacy of AML therapy.

As is usually the case in science, embarking on our journey of discovery has left us pleasantly surprised by the direction it is taking us. Future directions of the field are provided by two excellent reviews in this topic. Kulkarni and Kale provide a detailed review of reported adhesion molecules in hematopoietic stem cells, and discuss physiological cues involved in their regulation, with special focus on extracellular vesicles (EVs) as communication tools between hematopoietic stem cells and the niche. Balise et al. focus on tetraspanins, a family of membrane scaffold proteins which compartmentalize membrane adhesion and signaling receptors as well as intracellular signaling molecules, providing a spatiotemporal organization of membrane-associated proteins to influence critical hematopoietic stem cell functions, such as quiescence, self-renewal, differentiation, adhesion, migration and signaling. Tetraspanins are also one of the most commonly found protein classes in EVs.

We will sign off with a sincere thank you to the reviewers who kindly devoted their time, in spite of the ongoing pandemic, to the rigorous vetting and enrichment of the submitted manuscripts. This topic would not have been possible without your effort.
